# Cortical and subcortical correlates of perceptual responses during sinusoidal galvanic vestibular stimulation: a BOLD fMRI study

**DOI:** 10.1007/s00221-026-07411-w

**Published:** 2026-10-15

**Authors:** Brendan McCarthy, Donggyu Rim, Gianni Sesa-Ashton, Lewis S Crawford, Luke A Henderson, Vaughan G Macefield

**Affiliations:** 1 Baker Heart and Diabetes Institute, 75 Commercial Road, Melbourne, VIC 3004 Australia; 2 Baker Department of Cardiometabolic Health, The University of Melbourne, Melbourne, VIC Australia; 3 Department of Neuroscience, Monash University, Melbourne, VIC Australia; 4 School of Medical Sciences (Neuroscience), Brain and Mind Centre, The University of Sydney, Sydney, NSW Australia

**Keywords:** fMRI, Nausea, sGVS, Vestibular perceptions, Vestibular system

## Abstract

The cortical and subcortical circuitry underpinning motion sickness is poorly understood. However, certain regions – including the nucleus of the solitary tract (NTS) and the parieto-insular vestibular cortex (PIVC) – are known to be involved in the generation of nausea. The PIVC, in particular, is believed to serve an important role in the processing of vestibular inputs. We have previously used low-frequency sinusoidal galvanic vestibular stimulation (sGVS) to induce perceptions of side-to-side motion, which also induces nausea in ~ 40% of people. While sGVS has been previously used with brain imaging to investigate vestibular pathways, those studies used shorter bouts of stimulation (~ 30 s) which did not induce nausea. Here, we have used functional magnetic resonance imaging (fMRI) to record blood-oxygen-level-dependent (BOLD) signal-intensity changes at baseline (5 min), during low-frequency sGVS for a sustained period of time (± 2 mA, 0.08 Hz, 100 cycles; ~21 min) and during a recovery period (9 min) in 17 participants. Surprisingly, however, only four participants reported incipient or overt nausea during stimulation. Nevertheless, two regions displayed BOLD signal-intensity changes associated with the long-form (as opposed to previously used short-form) stimulation: the nucleus accumbens and locus coeruleus. Though sensations of nausea were not as apparent as expected (perhaps due to the necessity of participants adopting a supine position with their heads fixed in place), these regions would appear to be more associated with a longer period of vestibular stimulation, perhaps required to evoke sensations of nausea.

## Introduction

Motion sickness is a complex response presenting with a diverse array of symptoms including cold sweating, facial pallor, and of course, nausea (Golding 2016). For those who are particularly afflicted, this nausea may even progress to an emetic response – that is, vomiting. In spite of how commonplace the malady is in the modern world (with transport, virtual reality and even space travel), motion sickness remains a field of research with many unknowns. This extends to its origins (Guedry et al. 1998; Yates et al. 1998; Cha et al. 2021), an individual’s level of susceptibility (Grunfeld and Gresty 1998; Golding 2006; Lamb and Kwok 2015) and, indeed, a common cortical pathway through which the nausea and vomiting response is processed. With this being said, a number of studies have identified the dorsal vagal complex – comprising of the nucleus of the solitary tract (NTS), area postrema and dorsal motor nucleus of the vagus nerve (DMX) – to play a key role in the process (Babic and Browning 2014; Chen et al. 2018; Zhong et al. 2021). The NTS itself is capable of acting as an integrative hub for nauseogenic information, sending and receiving signals that may eventually lead to emesis.

The dorsal vagal complex is not the only region to be involved in the motion sickness response, however, as involvement of the vestibular system is also well established (Bertolini and Straumann 2016; Golding 2016; Cha et al. 2021). The vestibular apparatuses – the otoliths (saccule and utricle) and semi-circular canals, sensitive to translational and rotational acceleration respectively – determine one’s sense of balance and spatial awareness, in conjunction with proprioceptive inputs (Cheng and Gu 2018) and, of course, vision (Wade and Jones 1997). Much of this processing takes place in the parieto-insular vestibular cortex (PIVC), comprising of the parietal operculum (PO), insula and retroinsular regions (Eickhoff et al. 2006; zu Eulenburg et al. 2012; Ibitoye et al. 2023).

Low-frequency (< 1 Hz) sinusoidal galvanic vestibular stimulation (sGVS; not to be confused with stochastic or “noisy” GVS), delivered bilaterally to the mastoid processes via surface electrodes, induces illusions of sway through entraining vestibular afferents to the sinusoidal waveform (Bent et al. 2006; Klingberg et al. 2015). In doing so, this stimulation has the further effect of inducing motion sickness-related nausea in some individuals (Klingberg et al. 2015; Javaid et al. 2019). Though sGVS has been used while performing functional magnetic resonant imaging (fMRI) of the brain to identify vestibular pathways (Lobel et al. 1998; Stephan et al. 2005; Becker-Bense et al. 2020), many of the past studies apply the stimuli for only brief periods of time (less than one minute) and no studies, to the best of the authors’ knowledge, have reported findings on the real-time nausea associated with the stimulation.

The present study has aimed to address this lack of real-time nausea imaging data and, acts in direct continuation from the limitations of our own recent work in low-frequency sGVS, which also failed to induce nausea in the MRI environment (McCarthy et al. 2025). As we previously suggested, the higher stimulation frequency (0.2 Hz for 60 cycles) and shorter duration (5 min) used in the previous study may have contributed to the absence of nausea. Therefore, the current study has used a lower stimulation frequency (0.08 Hz) and longer duration (100 cycles; ~ 21 min), which we know induces nausea in about half of the participants (McCarthy et al. 2023). Given the much higher prevalence of nausea seen in McCarthy et al. (2023) and previous reports that lower frequencies of stimulation are more nauseogenic (James et al. 2010; Javaid et al. 2019), it was hypothesised that the lower-frequency stimulation would induce nausea in a significant proportion of participants. Furthermore, it was reasoned that this nausea would be most associated with changes in blood-oxygen-level-dependent (BOLD) signal intensity in the PIVC and NTS.

## Methods

### Subjects and ethics

20 participants were recruited, in line with McCarthy et al. 2025; however three were excluded from the data, as they had incomplete scans due to: (i) claustrophobia, (ii) image quality interference from a dental implant, or (iii) abnormal brain morphology. Experiments were performed on the remaining 17 healthy adults (11 females and six males; age: 21–31 years, mean ± standard deviation (SD), 24.1 ± 2.8 years; height: 151–180 cm, 163.3 ± 9.4 cm; weight: 45–88 kg, 60.7 ± 11.3 kg). Exclusion criteria included any contraindications to the MRI, as well as ongoing diagnosed vestibular disorders. The study received approval from the Human Research Ethics Committee (HREC) of the Alfred Hospital (HREC approval 62155) and was endorsed by Governance of the Baker Heart and Diabetes Institute. All participants provided informed written consent, conforming to the Declaration of Helsinki.

### Stimulation procedure

Square, magnetic resonance imaging (MRI)-compatible, 50 mm, Ag/AgCl surface electrodes (ConMed, Utica, USA) were placed on the mastoid processes on both sides of the head at the beginning of each experiment. Each participant then had gauze wrapped and securely fastened over the electrodes and around their heads in order to optimise electrode-to-skin contact.

While in a seated position prior to entering the MRI, each participant was subjected to two cycles of the same sinusoidal stimulus (± 2 mA at 0.08 Hz) that would be administered during the scanning procedure. This was done for the purpose of determining tolerance to the stimulus as delivered through an isolated constant-current stimulator (Linear Stimulus Isolator, World Precision Instruments, Florida, USA) and controlled by a PowerLab system (ADInstruments, Sydney, Australia) connected to a laptop computer. After testing, participants were questioned regarding their tolerance (do they believe they will be able to tolerate the stimulus for the duration of the experiment? ) and whether or not they perceived any motion or nausea.

Following the establishment of tolerance, participants were asked to lie on the MRI bed and were supplied with earplugs and cushioning pads on either side of their heads to lessen the MRI noise and movement of the head, respectively. Furthermore, participants were given an emergency buzzer to stop the experiment at any time. They were then subsequently positioned inside the bore of the 3 Tesla (3T) MRI machine (64-channel head/neck coil, MAGNETOM Prisma, Siemens Healthcare, Erlangen, Germany) to begin the experiment.

Firstly, a T1-weighted anatomical scan (MPRAGE sequence; single shot acquisition, repetition time (TR): 2300 ms, echo time (TE): 2.54 ms, voxel size: 0.8 mm isotropic, 192 slices) was acquired before the start of a single 35-minute echo-planar image with BOLD contrast fMRI scan (TR: 2500 ms, TE: 40 ms, voxel size: 2.2 mm isotropic, interleaved acquisition, 70 axial slices, 840 volumes, motion-corrected). The ~ 21-minute stimulus protocol (± 2 mA, 0.08 Hz, 100 cycles) was delivered during the BOLD fMRI scan, preceded by a five-minute baseline period and followed by an approximately nine-minute recovery period.

### Perception reporting

Prior to entering the MRI, participants were instructed to indicate the onset and conclusion of any nausea they may feel with a raise of their foot (situated outside of the MRI bore) for approximately two seconds. With this action an investigator would be able to note the start time and duration of any nausea and could then correlate it to the corresponding fMRI volumes.

Upon conclusion of the scanning procedure, participants were asked a series of questions concerning the stimulus and any perceptions they may have felt during the scan. The questions were as follows:

- Did you feel a sense of sway during the stimulation? If so, please describe it.
- Did you feel as if the sway was stronger towards one side?
- Did you feel as if the sway dissipated over time?
- Did you feel nauseous during the scanning procedure? If so, how intense was the feeling on a scale of 1 (minimal nausea) to 10 (maximal nausea; felt as if you would vomit)?
- Did you have any other perceptions or sensations during the scanning procedure? If so, what were they?

### Image pre-processing

Brain scan images were pre-processed and analysed using the statistical and parametric mapping software (SPM12) of Matlab R2022b (MathWorks, Massachusetts, USA). Due to spin saturation effects, the first five volumes of the fMRI series were discarded. The 835 remaining volumes were corrected for slice timing and motion. These were then fitted with a linear model of the global signal (LMGS) for detrending in order to remove global signal changes. Furthermore, a linear modelling of realignment parameters (LMRP) procedure (Macey et al. 2004) was applied to counteract the six-parameter-movement-related (i.e. three rotational and three translational movements) signal changes during the long recording period. The fMRI images of each participant were then coregistered to their corresponding T1-weighted images, which in turn were spatially normalised to the standard Montreal Neurological Institute (MNI) space via the diffeomorphic anatomical registration through exponentiated lie algebra (DARTEL) template. The fMRI image sets were spatially normalised and subsequently smoothed using a 5 mm full width at half maximum (FWHM) Gaussian kernel. Prior to the smoothing, however, fMRI images were also normalised to a spatially unbiased infra-tentorial (SUIT) template in MNI space. This was done to more precisely analyse the brainstem and cerebellum using the Matlab R2022b SUIT toolbox (Diedrichsen 2006). For these brainstem-only image sets, spatial smoothing was set to 3 mm.

To determine significant signal-intensity changes, a box-car model convolved with a haemodynamic delay function compared the stimulation period to both the baseline and the recovery period. The resulting statistical maps for both the wholebrain and brainstem-only images were then placed into second-level full factorial analyses with stimulus types added as factors of interest. To facilitate direct comparison with our prior study (McCarthy et al. 2025) the same thresholds for significant clusters were used. The statistical threshold was set to *P* < 0.001 (uncorrected), with the clusters further being defined by a threshold of 40 contiguous voxels for the cortical and 5 voxels for the brainstem-only analysis, to reduce the incidence of type 1 errors.

## Results

### Participant-reported perceptions

Each of the 17 participants reported a sense of sway during the sGVS protocol. Fourteen of these sensations were described as “side-to-side” motion, but one participant indicated that the sway was closer to a *combination* of “up-and-down” and “side-to-side” movement, while another two participants indicated that the perception of movement was more “rotational” or “spinning”. However, only two of the 17 participants reported feeling nauseous, with both describing the intensity of the nausea with a rating of 5 out of 10. Two other participants described a sensation of “pre-nausea” and “being on the brink of nausea”, respectively. In these cases, the participants indicated that they did not feel nauseous, per se, but rather from past experience that they were *close* to nausea. Unprompted, the participants further commented that, had they been able to move around or were sitting up, they felt the nausea would have been present. This sample size was not robust enough to undergo further statistical analysis.

Separate from any nauseogenic sensations, four participants reported that the sway was leftward dominant, while one reported a more rightward dominance – each of the other 12 reported equal sway on both sides. Nine participants indicated that the sway they felt dissipated over the length of the experiment. Interestingly, however, two participants stated that the strength of the perception of sway *increased* towards the end of the stimulation protocol. Five participants described the sense of sway during the scanning procedure as less pronounced than the sway experienced during the trial stimulation period conducted while sitting up, prior to entering the MRI. Finally, two participants reported a metallic taste in their mouths during stimulation, though this can likely be attributed to off-target stimulation of the taste afferents within the chorda tympani running through the inner ear (Bull 1965).

### Quality check for movement

Given the long scanning protocol, quality control checks were conducted for head movement in each experiment in each of the six directions. The axis, range of movement and mean movement across participants for each direction were as follows: X-axis: -0.45–0.56 mm (mean: -0.038 ± 0.24 mm); Y-axis: 0.01–0.55 mm (mean: 0.24 ± 0.17 mm); Z-axis: -1.20–0.51 mm (mean: -0.22 ± 0.47 mm); Pitch: -0.012–0.018 mm (mean: 0.0036 ± 0.0098 mm); Roll: -0.017–0.013 mm (mean: 0.00055 ± 0.0067 mm); Yaw: -0.01–0.01 mm (mean: -0.00013 ± 0.0047 mm).

### BOLD signal changes between baseline and sGVS

Clusters with significant signal-intensity changes, both increases and decreases, during sGVS when compared to the baseline resting period before stimulation are listed in Table 1. Several cortical regions displayed signal-intensity changes, but only one brainstem cluster was identified in this analysis.

**Table 1:** Regions with significant signal-intensity changes between baseline and stimulation

	Region	T-score	X	Y	Z	Cluster Size	Signal Change
Increases	Cortical Region						
	Left Anterior Insula	6.03	-30	6	9	518	0.41 ± 0.08
	Left Premotor Cortex	5.51	-39	6	48	118	0.20 ± 0.04
	Left Parietal Operculum	5.49	-45	-31.5	21	148	0.28 ± 0.05
	Left Posterior Cingulate Cortex	4.99	-12	-28.5	42	57	0.22 ± 0.04
	Subcortical Region						
	Medial Cerebellar Cortex (Lobule VIIb)	6.41	0	-69	-28.5	90	0.27 ± 0.05
	Left Cerebellar Cortex (Lobule VIIIa)	6.39	-22.5	-67.5	-48	239	0.24 ± 0.04
	Right Cerebellar Cortex (Lobule VI)	4.91	19.5	-72	-27	63	0.23 ± 0.05
	Left Cerebellar Cortex (Lobule VI)	4.70	-22.5	-66	-24	136	0.29 ± 0.06
	Left Cerebellar Cortex (Lobule V)	4.58	-31.5	-60	-28.5	41	0.30 ± 0.06
	Brainstem Region						
	Left Locus Coeruleus	4.81	-9	-38	-30	12	0.13 ± 0.03
Decreases	Cortical Region						
	Right Temporal Pole	7.62	40.5	9	-24	60	-0.29 ± 0.04
	Right Temporal Pole	6.46	43.5	10.5	-42	598	-0.35 ± 0.05
	Right Primary Motor Cortex/Primary Somatosensory Cortex	6.07	43.5	-7.5	37.5	186	-0.25 ± 0.04
	Right Transverse Temporal Gyrus	5.86	61.5	-15	0	155	-0.31 ± 0.05
	Right Occipital Cortex	5.24	45	-73.5	15	259	-0.22 ± 0.03
	Right Occipital Cortex	5.18	19.5	-82.5	19.5	435	-0.27 ± 0.05
	Left Subgenual Anterior Cingulate Cortex	5.05	-7.5	24	-6	111	-0.28 ± 0.05
	Right Primary Motor Cortex	4.99	34.5	-15	55.5	90	-0.15 ± 0.03
	Right Occipital Cortex	4.96	34.5	-79.5	10.5	102	-0.26 ± 0.05
	Right Premotor Cortex	4.85	31.5	6	54	101	-0.24 ± 0.05
	Subcortical Region						
	Right Nucleus Accumbens	6.01	9	27	0	182	-0.44 ± 0.08
	Right Nucleus Accumbens	5.00	21	21	-15	185	-0.39 ± 0.08
	Brainstem Region						
	N/A						

Regions displaying a significant blood-oxygen-level-dependent (BOLD) signal-change during stimulation when compared to the baseline resting period are listed. Both increases and decreases in signal-intensity are displayed, along with the T-score, location, cluster size and signal change. Coordinates to each cluster are listed with reference to Montreal Neurological Institute (MNI) space. Signal change is displayed as mean ± standard error of mean

#### Cortical and subcortical changes

**Fig. 1:**
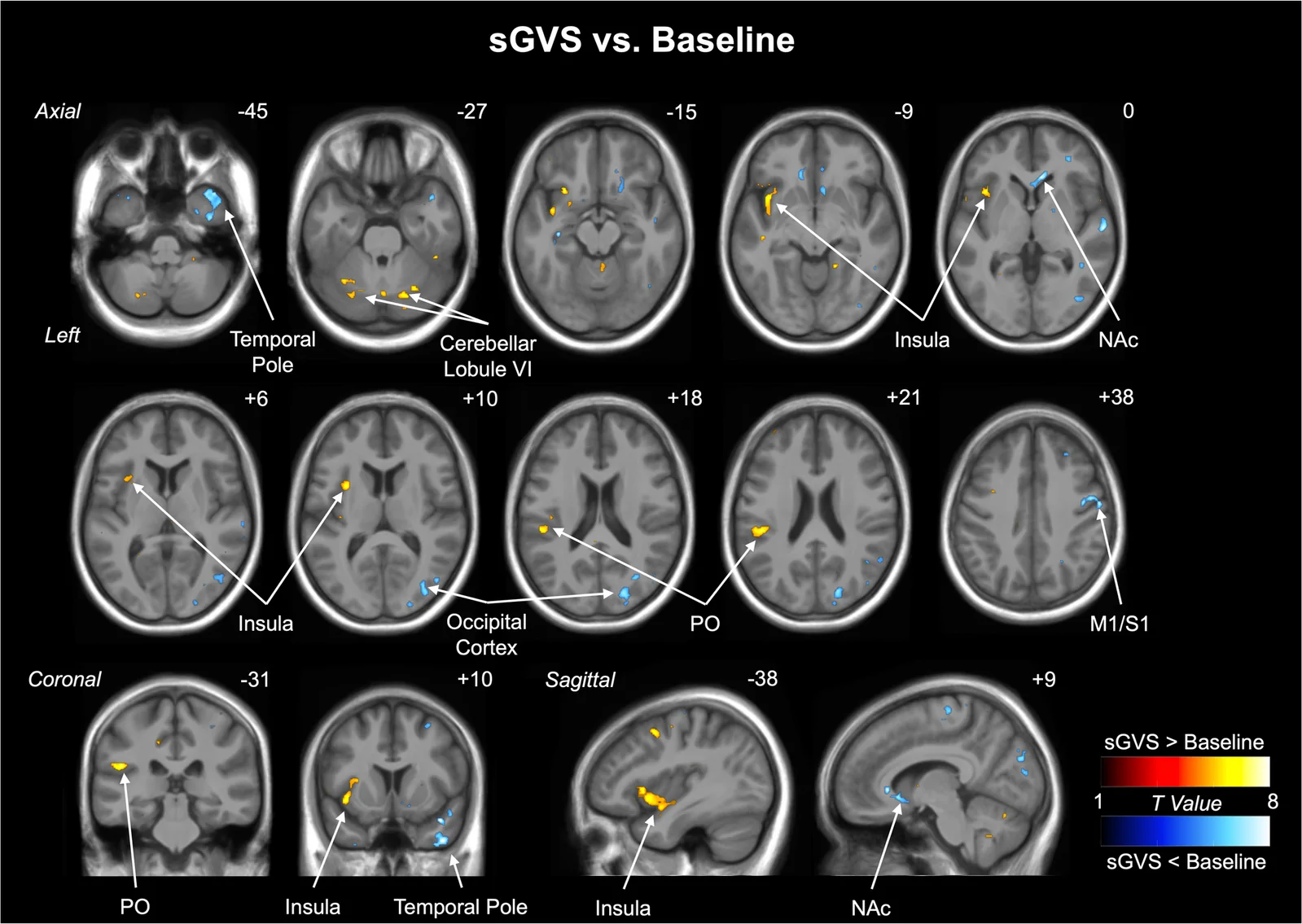
Cortical and subcortical signal-intensity changes during sGVS relative to baseline. Significant signal-intensity changes during sinusoidal galvanic vestibular stimulation (sGVS) when compared to the baseline resting period are displayed. The warm colour scale indicates an increase in blood-oxygen-level-dependent (BOLD) signal change from baseline, while the cool colour scale indicates a signal-decrease. Significant clusters are overlaid onto a mean T1-weighted anatomical image set. Slice locations in Montreal Neurological Institute space are indicated at the top right of each slice. *M1/S1: primary motor/somatosensory cortex; NAc: nucleus accumbens; PO: parietal operculum*

Prolonged low-frequency stimulation of the vestibular apparatuses resulted in signal-intensity increases unilaterally in the left anterior insula, parietal operculum (PO) and motor cortex. Further increases were found in the cerebellar cortex bilaterally. In contrast, decreases were found predominantly on the right side in and around the primary motor and somatosensory cortices, the nucleus accumbens (NAc), the temporal pole and the occipital cortex. One decrease was found on the left side, however, in the subgenual anterior cingulate cortex (subACC). Cortical and subcortical regions with significant signal-intensity changes are displayed in Fig. 1. Given the scope of the voxel space used in the current study, in lieu of multiple comparisons testing, clusters described in further detail within the discussion section below survived small volume correction (6 mm radius) with reference to Becker-Bense et al. (2020) and zu Eulenburg et al. (2012) for the PIVC and Napadow et al. (2013) for the locus coeruleus.

#### Brainstem changes

**Fig. 2:**
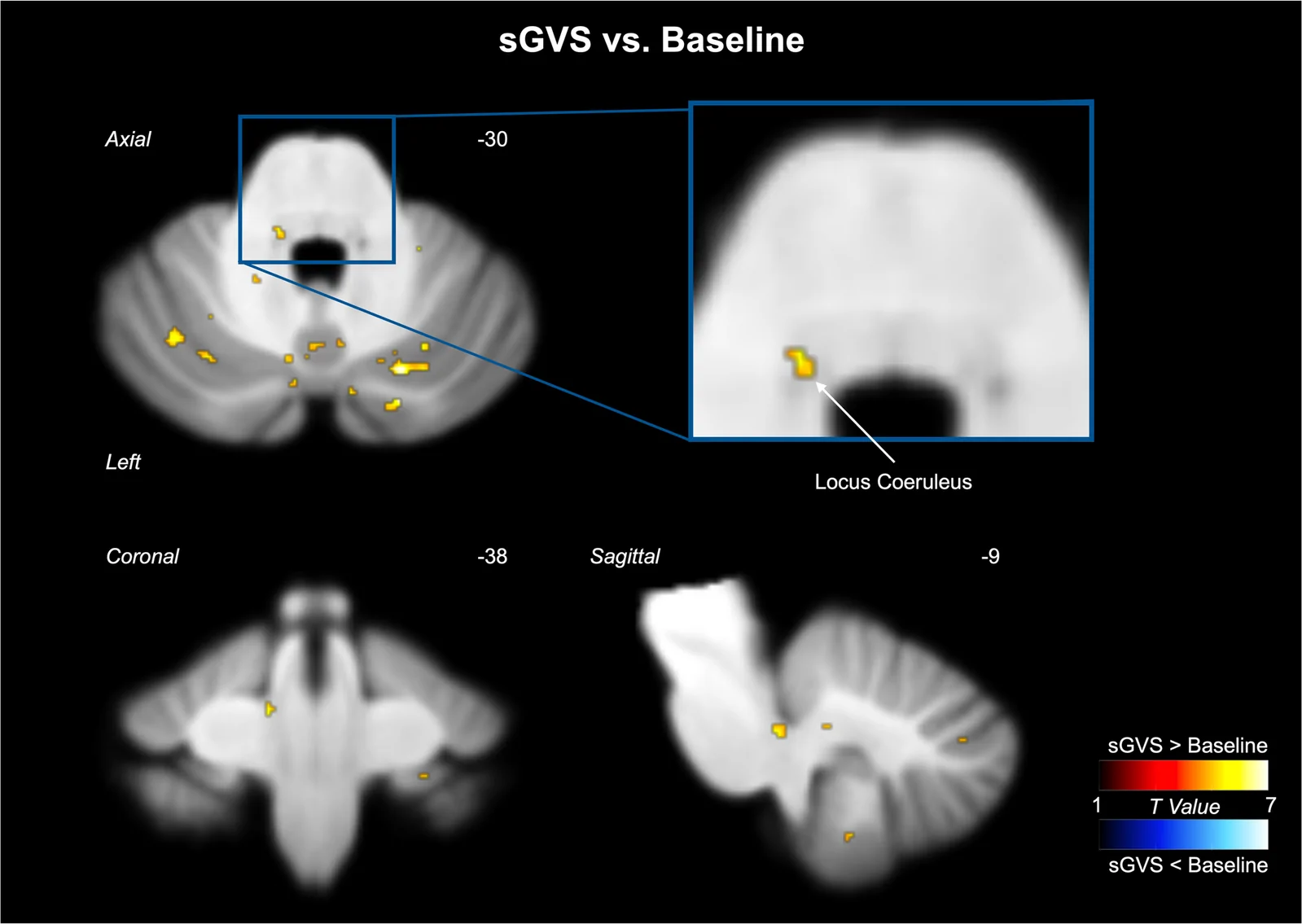
Brainstem signal-intensity changes during sGVS relative to baseline. Brainstem regions with significant signal-intensity changes during sinusoidal galvanic vestibular stimulation (sGVS) when compared to the baseline resting period are displayed. The warm colour scale indicates an increase in blood-oxygen-level-dependent (BOLD) signal change from baseline, with only the locus coeruleus identified through this analysis. A cool colour scale indicates signal-decrease; however, no regions were identified to have signal decreases in the brainstem during sGVS. Significant clusters are overlaid onto a mean T1-weighted anatomical image set. Slice locations in Montreal Neurological Institute space are indicated at the top right of each slice. No brainstem regions displayed significant signal changes when comparing sGVS to the recovery period

Only one brainstem area displayed significant signal intensity increases from baseline during stimulation, and this was around the left locus coeruleus (Fig. 2). No brainstem areas showed significant decreases in signal-intensity in this comparison.

### BOLD signal changes between sGVS and recovery

Clusters showing significant increased and decreased signal-intensity changes during sGVS when compared to the recovery period after stimulation are listed in Table 2. Several regions displayed signal-intensity increases during stimulation, but only one cluster indicating a decrease in signal-intensity was identified in the cortex. No signal-intensity changes were found in the brainstem.

**Table 2:** Regions with significant signal-intensity changes between stimulation and recovery

	Region	T-score	X	Y	Z	Cluster Size	Signal Change
Increases	**Cortical Region**						
	Left Secondary Motor Cortex	7.99	-15	-28.5	39	327	0.17 ± 0.03
	Left Posterior Insula	6.41	-33	-21	9	73	0.23 ± 0.04
	Left Supplementary Motor Area	5.98	-22.5	1.5	46.5	166	0.11 ± 0.02
	Left Parietal Operculum	5.74	-55.5	-37.5	31.5	240	0.19 ± 0.03
	Left Parietal Operculum	5.41	-55.5	-16.5	18	65	0.20 ± 0.04
	Left Parietal Operculum	5.36	-37.5	-13.5	31.5	76	0.13 ± 0.03
	Left Parietal Operculum	5.29	-57	-10.5	9	45	0.29 ± 0.06
	Left Dorsolateral Prefrontal Cortex	5.19	-27	40.5	34.5	49	0.16 ± 0.03
	Left Parietal Operculum	5.14	-60	3	13.5	52	0.20 ± 0.04
	Left Primary Somatosensory Cortex	5.06	-52.5	-19.5	30	88	0.17 ± 0.04
	Left Parietal Operculum	4.10	-42	-34.5	21	67	0.19 ± 0.03
	Right Posterior Insula	4.86	33	-18	10.5	78	0.19 ± 0.03
	Left Primary Motor Cortex	4.84	-36	-4.5	57	72	0.19 ± 0.04
	Left Premotor Cortex	4.53	-40.5	13.5	49.5	90	0.18 ± 0.03
	Left Supplementary Motor Area	4.52	-19.5	-1.5	67.5	53	0.22 ± 0.05
	Left Primary Motor Cortex	4.32	-25.5	-25.5	64.5	48	0.22 ± 0.05
	**Subcortical Region**						
	Right Thalamus	5.50	10.5	-22.5	6	40	0.17 ± 0.03
	Left Cerebellar Cortex (Lobule VIIb)	5.22	-13.5	-73.5	-54	43	0.23 ± 0.04
	Medial Cerebellar Cortex (Lobule IX)	4.92	1.5	-54	-40.5	42	0.14 ± 0.03
	**Brainstem Region**						
	N/A						
Decreases	**Cortical Region**						
	Right Temporal Pole	8.18	34.5	4.5	-30	133	-0.22 ± 0.03
	**Subcortical Region**						
	N/A						
	**Brainstem Region**						
	N/A						

Regions displaying a significant blood-oxygen-level-dependent (BOLD) signal-change during stimulation when compared to the recovery period are listed. Both increases and decreases in signal-intensity are displayed, along with the T-score, location, cluster size and signal change. Coordinates to each cluster are listed with reference to Montreal Neurological Institute (MNI) space. Signal change is displayed as mean ± standard error of mean

#### Cortical and subcortical changes

**Fig. 3:**
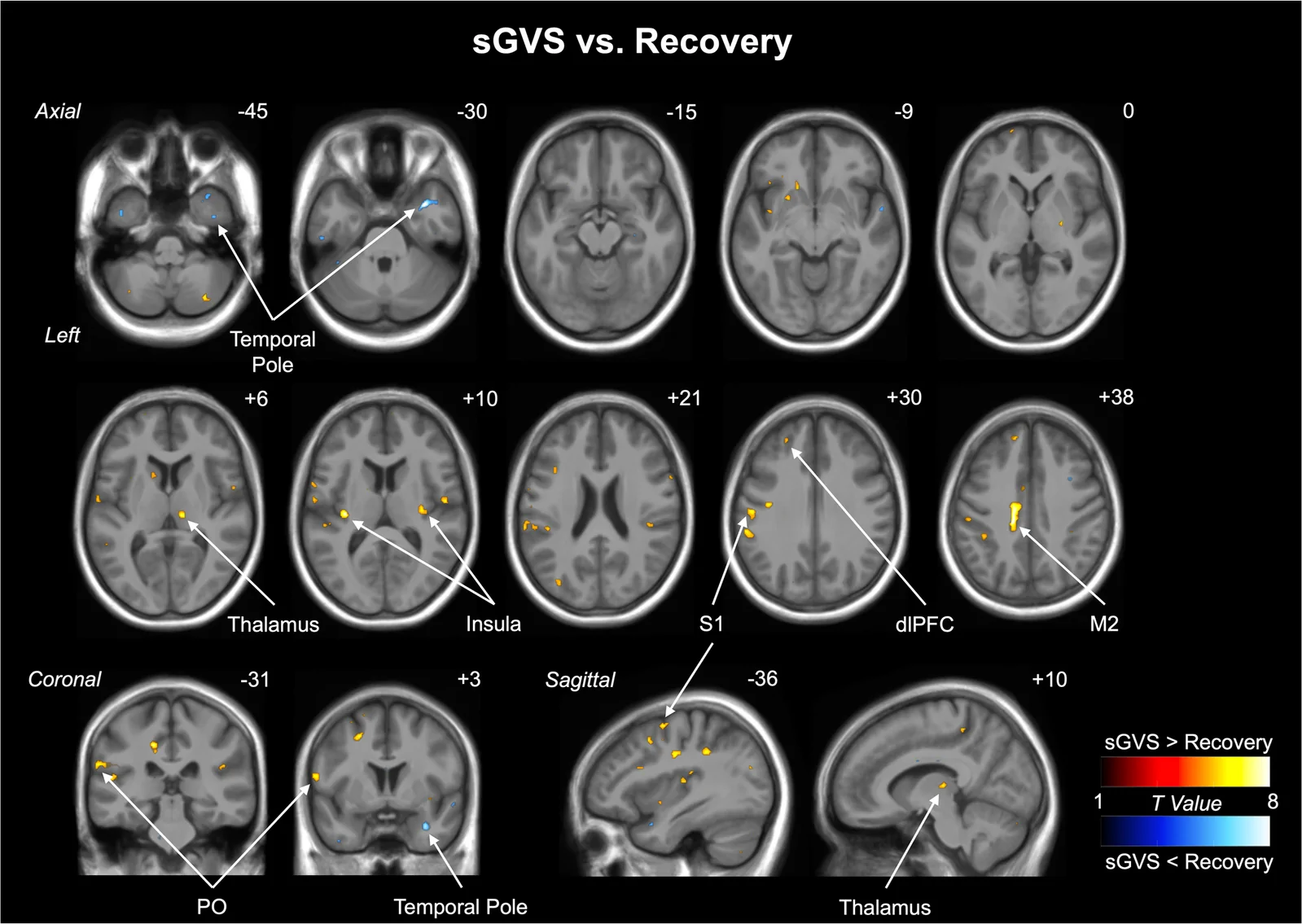
Cortical and subcortical signal-intensity changes during sGVS relative to the recovery period. Significant signal-intensity changes during sinusoidal galvanic vestibular stimulation (sGVS) when compared to the recovery period after stimulation are displayed. The warm colour scale indicates an increase in blood-oxygen-level-dependent (BOLD) signal change from baseline, while the cool colour scale indicates a signal-decrease. Significant clusters are overlaid onto a mean T1-weighted anatomical image set. Slice locations in Montreal Neurological Institute space are indicated at the top right of each slice. *dlPFC: dorsolateral prefrontal cortex; M2: secondary motor cortex; PO: parietal operculum; S1: primary somatosensory cortex*

When compared to the recovery period, sGVS produced increases in signal-intensity bilaterally in the posterior insula. Alongside this, unilateral increases were found in the left PO, motor and somatosensory cortices, dorsolateral prefrontal cortex (dlPFC) and cerebral cortex, with one unilateral signal-increase found on the right side within the thalamus. Contrary to stimulation vs. baseline, the only signal-intensity decrease during stimulation vs. recovery was found in the right temporal pole (Fig. 3).

#### Brainstem changes

No brainstem region displayed an increase or decrease in signal-intensity to the set thresholds during stimulation when compared to the recovery period.

## Discussion

Contrary to our hypothesis, implementing the low-frequency, long-duration stimulation protocol of McCarthy et al. (2023) – sGVS at 0.08 Hz for 100 cycles, ~ 21 min – did not significantly increase the incidence of nausea from our previous fMRI study (McCarthy et al. 2025) using a higher frequency but shorter duration of stimulation (0.2 Hz for 60 cycles; 5 min). While we acknowledge that the 5-minute baseline, 21-minute stimulation and 9-minute rest are unusual blocks of time for an MRI protocol, the timings were chosen based on our previous papers (McCarthy et al. 2023, 2025). The MRI parameters were the same as McCarthy et al. (2025) and the stimulation parameters were the same as McCarthy et al. (2023) (and other low-frequency sGVS studies conducted within our laboratory (Klingberg et al. 2015; Javaid et al. 2019). In theory, had the stimulus produced a significant amount of nausea, this would have facilitated comparisons between studies. Though the original objectives of the present study were not met, why this was so warrants discussion, and reasons for these findings will be elaborated upon below. While the expected incidence of nausea did not occur, the present study has, however, identified several brain regions including the PIVC, somatosensory and motor cortices, NAc and locus coeruleus to be intrinsically linked to this form of vestibular stimulation. Therefore, in shifting analysis away from nausea to comparisons between long- and short-form vestibular stimulation, the present study can still shed some light on cortical vestibular processing. How the aforementioned regions compare to those identified through the sGVS component of McCarthy et al. (2025) and, indeed, to other studies in the field will also be discussed in the following section.

### sGVS-induced nausea

Given the initial primary aim and subsequent outcome of the study, it is perhaps most important to first discuss participant-reported sensations of nausea. Excluding the two participants who described the sensation of “approaching nausea”, only two of the 17 participants reported overt nausea from the vestibular stimulation. While an unexpected result considering the nausea incidence in previous low-frequency sGVS studies from our laboratory (Klingberg et al. 2015; Javaid et al. 2019; McCarthy et al. 2023), these studies differ from the present one in a key factor: their orientation relative to gravity. The prior experiments reporting sGVS-induced nausea notably have participants in a seated position. This differs markedly from the supine position participants must adopt for MRI scanning. As such, it is important to discuss the axis of rotation. When seated or standing, the bilateral, bipolar sGVS generally induces perceptions of self-motion in the roll axis – hence the “swaying” sensation (Austin et al. 2026). In this situation, the perceived motion is tilt about the horizontal axis of the Earth, producing otolithic sensory conflict – one of the prevailing theories for the generation of motion sickness (Yates et al. 1998). However, when a person is lying supine (again, as they must be for a conventional MRI scan at 3T; upright MRI machines have low field strengths unsuitable for detailed imaging of the brain), the roll axis sway is now around the vertical axis of the Earth. Due to this, the otoliths are not subjected to the same degree of sensory conflict, as the tilt is in line with the graviceptor signals (Mittelstaedt 1996; von Gierke and Parker 1993). It is in this reason that we may find the primary cause for the lack of nausea induced by the otherwise highly nauseogenic sGVS protocol.

Furthermore, as we all know from experience, when we feel nauseous one of the first things we tend to do is lie down. This has even been proven to have significant effects on mitigating the symptoms of nausea (Golding et al. 1995). Interestingly, it has also been shown that restricting head movements – another unavoidable aspect of brain imaging – contributes significantly towards the prevention of motion sickness (Golding et al. 2003, 2006). Both of these reasons, as well, may have contributed towards the deficit in nausea generation.

Another aspect to discuss, however, are the transient vertigo-like perceptions that are induced in participants as they enter and exit the MRI bore. These perceptions are much more pronounced at stronger field intensities, such as 7T, but are nevertheless present at 3T as well (Theysohn et al. 2014). It is unlikely that this phenomenon, attributable to magnetic field interaction with the vestibular apparatus, would impact the results obtained, given that even at 7T perceptions subside within one minute (Mian et al. 2013). However, it is important to mention this factor, even if just to refute its involvement.

It is also important to discuss the possible influence of the menstrual cycle and individual susceptibility, in general, to the observed lack of a nauseogenic response, given that 11 of our 17 participants were female. Prior studies have shown fluctuating motion sickness susceptibility corresponding to the menstrual cycle in women (Golding et al. 2005; Matchock et al. 2008). Moreover, many other factors are known to influence susceptibility in both sexes (Grunfeld and Gresty 1998; Golding 2006; Lamb and Kwok 2015). Controlling for these variables by timing experiments around the menstrual cycle (or at the very least, taking note) and implementing a questionnaire for susceptibility may have had positive influences on the results of this study. With this being said, it is worth noting that our prior studies reporting the prevalence of nausea with low-frequency sGVS (Klingberg et al. 2015; Javaid et al. 2019; McCarthy et al. 2023) also did not control for these factors. As such, the largest difference between studies remains that caused by the MRI environment – participants lying down with their heads fixed in place.

One may assume that these MRI factors contribute towards the literature gap which exists surrounding sGVS-induced nausea concurrent to brain imaging. Yet, in taking a closer look at the methods of stimulation used by the past MRI studies, stimulation time would appear to be a key factor, too. In seeking to identify brain regions and networks associated with sGVS, each of the studies by Lobel et al. (1998), Stephan et al. (2005) and Becker-Bense et al. (2020) implemented stimuli in blocks of ~ 30 s. While effective for addressing the aims of each study in turn, this short duration would not allow enough time for nausea to develop. This does, however, present another means by which the present study can compare results to the studies before it – cortical regions associated with long-form stimulation rather than short.

### Cortical regions associated with prolonged sGVS

In line with our hypothesis, comparing the stimulation to the baseline resting period prior to sGVS saw increases in signal-intensity occurring within the insula and PO. This result is not surprising given the well-established literature base showing these regions of the PIVC to be pivotal in the vestibular processing pathway (Eickhoff et al. 2006; zu Eulenburg et al. 2012; Ibitoye et al. 2023). What is surprising, however, is the predominantly left-sided activation patterns within these regions. It has been established that PIVC activation is related to handedness, with right handers having right PIVC dominance, and left handers having left dominance (Dieterich and Brandt 2018). Unfortunately, handedness was not recorded in the current study, so how this may have impacted the results obtained cannot be conclusively stated. Had a great proportion of the participants been left-handed, the left PIVC activation patterns would make sense. However, this is incredibly unlikely given the proportion of right-handers in the general population (~ 90%). It is more likely due to discrepancies in methodology. Stimulation was administered in a binaural, bipolar fashion in our study, yet was delivered via caloric irrigation, auditorily or with direct currents in work showing the ipsilateral dominance (Dieterich et al. 2003; Fink et al. 2003; Brandt and Dieterich 2015). Alternatively, it may also be important to note the work by Craig (2005, 2015). The author argues that the left anterior insula cortex, as opposed to the right, is associated with nausea and, more broadly, disgust. This draws from the evidence that there is lateralisation of autonomic control within the brain, with the right being more important for controlling sympathetic outflow, and the left parasympathetic outflow. This would then lend to left-sided insula activation with the parasympathetic connections of disgust. Indeed, Oppenheimer et al. (1992) showed that electrical stimulation of the right insula in humans increases blood pressure and heart rate while the opposite occurs with stimulation of the left. Moreover, right-sided insular strokes lead to a greater incidence of cardiac arrythmias (Oppenheimer 1994; Fontes et al. 2024).

In contrast to our initial hypothesis though, the stimulation period saw no changes in signal-intensity within the NTS. Due to the lack of pronounced nausea, however, a result such as this is not entirely unexpected. The NTS plays more of a role in the emetic pathway, responding and contributing towards a visceral response (Zhong et al. 2021). Accordingly, had the stimulation produced such an effect in a large proportion of the participants, the results may have changed to include the NTS.

Perhaps more interestingly, therefore, is the significant signal-intensity decrease seen in the NAc during baseline to stimulation comparison. As discussed previously (McCarthy et al. 2025), the NAc is not commonly reported in studies involving vestibular stimulation. However, this region was deemed to be possibly key to the inhibitory interaction on vestibular illusions seen in our last study (McCarthy et al. 2025) and again was shown to be an important player in the present study. While the stimulation used in our previous study was shorter in duration than that used here, both are still orders of magnitude longer than prior sGVS brain imaging work (Lobel et al. 1998; Stephan et al. 2005; Becker-Bense et al. 2020). It may very well be that the NAc only builds association with vestibular stimulation over longer periods of time. In addition, it is well known that the NAc is involved in encoding of pleasantness, with decreases in BOLD signal-intensity occurring when participants are exposed to unpleasant images (Costa et al. 2010), for example. It may be that the sGVS also induces some degree of unpleasantness, though this was not asked of the participants.

While sGVS-induced nausea has not been explored in the context of fMRI before, it is important to note that nausea has been successfully induced during brain imaging. In their studies, Napadow et al. (2013) and Ruffle et al. (2019) identified several brain areas associated with nausea through a visual paradigm. These areas included the insular and somatosensory cortices with increasing nausea, and the amygdala and putamen preceding nausea. These latter two regions were not identified in the present study, though statistical thresholds may account for this. Due to the very small number of participants who experienced nausea, associations with more nausea-specific regions would likely be weaker in nature, if seen at all. As such, the high statistical thresholds used in the present study may have filtered out any low-level BOLD signal changes, including potential putamen and amygdala activations.

Importantly, however, Napadow et al. (2013) also noted signal increases in the locus coeruleus, as we did in the present study. This is all the more important when considering the fact that the other fMRI studies using sGVS (Lobel et al. 1998; Stephan et al. 2005; Becker-Bense et al. 2020), including our own (McCarthy et al. 2025), did not identify any signal changes in this brainstem region. Through Napadow et al. (2013) there is an established basis for locus coeruleus involvement in motion sickness and, as such, this area’s involvement in the present study, as opposed to the others, implies that the stimuli were perhaps more “nauseogenic” in nature than first thought. This is not to say that the stimuli generated the feeling of nausea any more than the two overt reportings, it merely serves to highlight the similarities between the studies. Of course, the participant-reported sensations of nausea did not significantly differ between this and our last sGVS MRI study (McCarthy et al. 2025), and a direct comparison between a nauseous and non-nauseous group was not possible. However, in the locus coeruleus we have evidence to suggest that the subcortical circuitry may have differed. Perhaps, for the reasons described above, nausea did not manifest in many of the participants, yet the underlying mechanisms for sGVS-induced nausea were still present. In fact, the locus coeruleus has been further implicated in this form of nausea in cats with sGVS being delivered intracranially through electrodes placed adjacent to the labyrinths bilaterally and post-mortem c-fos protein labelling (Balaban et al. 2014). Moreover, this brain region is known to have direct connections with the vestibular nuclei (Balaban 1996). Therefore, the locus coeruleus might have significance in sGVS-induced nausea (or at least longer-form sGVS), but not the vestibular perceptions of motion.

In comparing sGVS to the recovery period, again the PIVC was found to have significant signal-intensity increases, as expected. Furthermore, this comparison saw significant signal-intensity increases within the motor cortex. These results are not surprising either, as sGVS is known to activate the vestibulomotor pathway (Cullen 2019; Cullen and Wang 2020). In essence, significant signal-intensity changes between stimulation and recovery did not reveal any abnormal or outstanding regions. When compared to the baseline vs. stimulation results, however, it is interesting to note that the NAc and locus coeruleus did not appear. Through this, it seems that perhaps the two regions somewhat sustain their BOLD signal-intensity changes past the stimulation period. Had they returned to baseline levels, it would be expected that they would appear once more as areas with significant signal change during sGVS. From this, we have evidence to support the idea that the circuitry involved in the processing of long-form sGVS (if, indeed, these regions constitute parts of the circuitry) may take a protracted period of time to return to baseline levels; just as they would appear to take a longer amount of time to show changes during stimulation (as they are not seen in shorter stimuli experiments described above).

### Limitations

The primary limitation of this work is, of course, the lack of overt nausea reporting. The initial aim was to induce sGVS-related nausea in participants with a prevalence similar to that seen in our previous work separate from the MRI (~ 36%) (McCarthy et al. 2023). Unfortunately, while this study has provided data on longer-duration sGVS and was able to compare these data with both previous data obtained within our laboratory (McCarthy et al. 2025) and previous studies in the field (Lobel et al. 1998; Stephan et al. 2005; Becker-Bense et al. 2020), the incidence of nausea was far lower than expected. Nevertheless, the two participants who reported incipient nausea may well have developed overt nausea had the stimulation been longer; we know empirically that nausea often starts with an uncertain feeling that builds up over time, though in the two who reported overt nausea this was comparatively quick. As discussed above, the relative absence of the nausea outcome can be attributed to unavoidable factors within the MRI environment such as lying down and being fixed in place, or even factors surrounding susceptibility (a prior motion sickness susceptibility assessment was also not conducted in the present study). Regardless of the cause, though, this does mean that the gap in the literature regarding real-time brain imaging during episodes of sGVS-induced nausea still remains. In light of these results, it may be worthwhile shifting away from using sGVS to induce motion sickness-related nausea, using instead a visual protocol more similar to that of Napadow et al. (2013) or (Ruffle et al. 2019). In doing so, this would address another of the major limitations of the study – that being the differences in sGVS and more natural forms of vestibular stimulation. While sGVS is capable of inducing nausea, it is not a form of vestibular stimulation one would come across in everyday life. Therefore, the cortical and subcortical activation patterns seen in this study must take into consideration this factor, especially when extrapolating to general motion sickness.

It is also important to note that the imaging protocol contains limitations as well. While the current protocol was used in order to directly compare to our previous work (as described above), it may have been more apt to change the scope of the imaging to focus more on emotional processing areas and the brainstem. Though the latter was more deeply investigated through the use of a SUIT template, specific targeting may have revealed further signal-intensity changes in areas such as the NTS and vestibular nuclei, however human brainstem imaging is somewhat constrained in viewing this region, especially at 3T.

Finally, as stated in the discussion, handedness was not recorded in the present study. As such, the lateralisation of the results must be interpreted with caution – especially since handedness and vestibular lateralisation have been shown to be quite robustly linked (Dieterich and Brandt 2018).

## Conclusions

Despite evidence to suggest that lower frequency, longer duration sGVS will induce a greater prevalence of nausea in the seated position, this did not translate into the MRI environment. The most probable cause for this finding is the fact that participants must lie supine with their heads fixed firmly in place to undergo brain imaging. That said, the long-form stimulation was still able to reveal BOLD fMRI signal-intensity changes within two outstanding regions: the NAc and locus coeruleus. It would seem that both these regions are more associated with longer periods of vestibular stimulation, rather than shorter stimulation protocols used previously, and may play a role in the underlying mechanisms of sGVS-induced nausea, regardless of the deficit of overt nausea symptoms.

## Data Availability

The data are available upon reasonable request.

## References

[CR1] Austin CR, Willett L, Clark TK (2026) Motion sensations, postural sway, and side effects for copolar galvanic vestibular stimulation. Exp Brain Res 244. 10.1007/s00221-026-07255-4 PMC12950039 41762227

[CR2] Babic T, Browning KN (2014) The role of vagal neurocircuits in the regulation of nausea and vomiting. Eur J Pharmacol 722:38–47. 10.1016/j.ejphar.2013.08.047 PMC3893663 24184670

[CR3] Balaban CD (1996) Vestibular nucleus projections to the parabrachial nucleus in rabbits: implications for vestibular influences on the autonomic nervous system. Exp Brain Res 108:367–381. 10.1007/BF00227260 8801117

[CR4] Balaban CD, Ogburn SW, Warshafsky SG, Ahmed A, Yates BJ (2014) Identification of neural networks that contribute to motion sickness through principal components analysis of fos labeling induced by galvanic vestibular stimulation. PLoS ONE 9:e86730. 10.1371/journal.pone.0086730 PMC3900607 24466215

[CR5] Becker-Bense S, Willoch F, Stephan T, Brendel M, Yakushev I, Habs M, Ziegler S, Herz M, Schwaiger M, Dieterich M, Bartenstein P (2020) Direct comparison of activation maps during galvanic vestibular stimulation: a hybrid H_2_[^15^O] PET-BOLD MRI activation study. PLoS ONE 15:e0233262. 10.1371/journal.pone.0233262 PMC7228124 32413079

[CR6] Bent LR, Bolton PS, Macefield VG (2006) Modulation of muscle sympathetic bursts by sinusoidal galvanic vestibular stimulation in human subjects. Exp Brain Res 174:701–711. 10.1007/s00221-006-0515-6 16721608

[CR7] Bertolini G, Straumann D (2016) Moving in a moving world: a review ion vestibular motion sickness. Front Neurol 7:14. 10.3389/fneur.2016.00014 PMC4753518 26913019

[CR8] Brandt T, Dieterich M (2015) Does the vestibular system determine the lateralization of brain functions? J Neurol 262:214–215. 10.1007/s00415-014-7548-8 25346066

[CR9] Bull TR (1965) Taste and the chorda tympani. J Laryngol Otol 79:479–493. 10.1017/S0022215100063969 14292248

[CR10] Cha YH, Golding JF, Keshavarz B, Furman J, Kim JS, Lopez-Escamez JA, Magnusson M, Yates BJ, Lawson BD (2021) Motion sickness diagnostic criteria: consensus document of the Bárány society. J Vestib Res 31:327–344. 10.3233/VES-200005 PMC9249300 33646187

[CR11] Chen MM, Xu LH, Chang L, Yin P, Jiang ZL (2018) Reduction of motion sickness through targeting histamine N-methyltransferase in the dorsal vagal complex of the brain. J Pharmacol Exp Ther 364:367–376. 10.1124/jpet.117.244475 29298819

[CR12] Cheng Z, Gu Y (2018) Vestibular system and self-motion. Front Cell Neurosci 22:456. 10.3389/fncel.2018.00456 PMC6262063 30524247

[CR13] Costa VD, Lang PJ, Sabatinelli D, Versace F, Bradley MM (2010) Emotional imagery: assessing pleasure and arousal in the brain’s reward circuitry. Hum Brain Mapp 31:1446–1457. 10.1002/hbm.20948 PMC3620013 20127869

[CR14] Craig AD (2005) Forebrain emotional asymmetry: a neuroanatomical basis? Trends Cogn Sci 9:566–571. 10.1016/j.tics.2005.10.005 16275155

[CR15] Craig AD (2015) How Do You Feel? An Interoceptive Moment with Your Neurobiological Self. Princeton University Press

[CR16] Cullen KE (2019) Vestibular processing during natural self-motion: implications for perception and action. Nat Rev Neurosci 20:346–363. 10.1038/s41583-019-0153-1 PMC6611162 30914780

[CR17] Cullen KE, Wang L (2020) Predictive coding in early vestibular pathways: implications for vestibular cognition. Cogn Neuropsychol 37:423–426. 10.1080/02643294.2020.1783222 PMC7704804 32619395

[CR18] Diedrichsen J (2006) A spatially unbiased atlas template of the human cerebellum. NeuroImage 33:127–138. 10.1016/j.neuroimage.2006.05.056 16904911

[CR20] Dieterich M, Brandt T (2018) The parietal lobe and the vestibular system. Handb Clin Neurol 151:119–140. 10.1016/B978-0-444-63622-5.00006-1 29519455

[CR19] Dieterich M, Bense S, Lutz S, Drzezga A, Stephan T, Bartenstein P, Brandt T (2003) Dominance for vestibular cortical function in the non-dominant hemisphere. Cereb Cor 13:994–1007. 10.1093/cercor/13.9.994 12902399

[CR21] Eickhoff SB, Weiss PH, Amunts K, Fink GR, Zilles K (2006) Identifying human parieto-insular vestibular cortex using fMRI and cytoarchitectonic mapping. Hum Brain Mapp 27:611–621. 10.1002/hbm.20205 PMC6871353 16281284

[CR22] Fink GR, Marshall JC, Weiss PH, Stephan T, Grefkes C, Shah NJ, Zilles K, Dieterich M (2003) Performing allocentric visuospatial judgments with induced distortion of the egocentric reference frame: an fMRI study with clinical implications. NeuroImage 20:1505–1517. 10.1016/j.neuroimage.2003.07.006 14642463

[CR23] Fontes MAP, dos Santos Machado LR, Viana ACR, Cruz MH, Nogueira ÍS, Oliveira MGL, Neves CB, Godoy ACV, Henderson LA, Macefield VG (2024) The insular cortex, autonomic asymmetry and cardiovascular control: looking at the right side of stroke. Clin Auton Res 34:549–560. 10.1007/s10286-024-01066-9 39316247

[CR24] Golding JF (2006) Motion sickness susceptibility. Auton Neurosci 129:67–76. 10.1016/j.autneu.2006.07.019 16931173

[CR25] Golding JF (2016) Motion sickness. Handb Clin Neurol 137:371–390. 10.1016/B978-0-444-63437-5.00027-3 27638085

[CR28] Golding JF, Markey HM, Stott JRR (1995) The effects of motion direction, body axis, and posture, on motion sickness induced by low frequency linear oscillation. Aviat Space Environl Med 66:1046–1051 8588793

[CR26] Golding JF, Bles W, Bos JE, Haynes T, Gresty MA (2003) Motion sickness and tilts of the inertial force environment: active suspension systems vs. active passengers. Aviat Space Environl Med 74:220–227 12650268

[CR27] Golding JF, Kadzere P, Gresty MA (2005) Motion sickness susceptibility fluctuates through the menstrual cycle. Aviat Space Environl Med 76:970–973 16235881

[CR29] Grunfeld E, Gresty MA (1998) Relationship between motion sickness, migraine and menstruation in crew members of a round the world yacht race. Brain Res Bull 47:433–436. 10.1016/S0361-9230(98)00099-9 10052571

[CR30] Guedry FE, Rupert AR, Reschke MF (1998) Motion sickness and development of synergy within the spatial orientation system. A hypothetical unifying concept. Brain Res Bull 47:475–480. 10.1016/S0361-9230(98)00087-2 10052577

[CR31] Ibitoye RT, Mallas EJ, Bourke NJ, Kaski D, Bronstein AM, Sharp DJ (2023) The human vestibular cortex: functional anatomy of OP2, its connectivity and the effect of vestibular disease. Cereb Cor 33:567–582. 10.1093/cercor/bhac085 PMC9890474 35235642

[CR32] James C, Stathis A, Macefield VG (2010) Vestibular and pulse-related modulation of skin sympathetic nerve activity during sinusoidal galvanic vestibular stimulation in human subjects. Exp Brain Res 202:29–298. 10.1007/s00221-009-2131-8 20041236

[CR33] Javaid A, Chouhna H, Varghese B, Hammam E, Macefield VG (2019) Changes in skin blood flow, respiration and blood pressure in participants reporting motion sickness during sinusoidal galvanic vestibular stimulation. Exp Physiol 104:1622–1629. 10.1113/EP087385 31468621

[CR34] Klingberg D, Hammam E, Macefield VG (2015) Motion sickness is associated with an increase in vestibular modulation of skin but not muscle sympathetic nerve activity. Exp Brain Res 233:2433–2440. 10.1007/s00221-015-4313-x 26025612

[CR35] Lamb S, Kwok KC (2015) MSSQ-short norms may underestimate highly susceptible individuals: updating the MSSQ-short norms. Hum Factors 57:622–633. 10.1177/0018720814555862 25977322

[CR36] Lobel E, Kleine JF, Bihan DL, Leroy-Willig A, Berthoz A (1998) Functional MRI of galvanic vestibular stimulation. J Neurophysiol 80:2699–2709. 10.1152/jn.1998.80.5.2699 9819274

[CR37] Macey PM, Macey KE, Kumar R, Harper RM (2004) A method for removal of global effects from fMRI time series. NeuroImage 22:360–366. 10.1016/j.neuroimage.2003.12.042 15110027

[CR38] Matchock RL, Levine ME, Gianaros PJ, Stern RM (2008) Susceptibility to nausea and motion sickness as a function of the menstrual cycle. Women’s Health Issues 18:328–335. 10.1016/j.whi.2008.01.006 PMC2525506 18485739

[CR39] McCarthy B, Datta S, Sesa-Ashton G, Wong R, Dawood T, Henderson LA, Macefield VG (2023) Top-down control of vestibular inputs by the dorsolateral prefrontal cortex. Exp Brain Res 241:2845–2853. 10.1007/s00221-023-06722-6 PMC10635918 37902866

[CR40] McCarthy B, Rim D, Sesa-Ashton G, Crawford LS, Dawood T, Henderson LA, Macefield VG (2025) Electrical stimulation of the dorsolateral prefrontal cortex inhibits vestibular signalling in humans: a BOLD fMRI study. Brain Stimul 18:627–639. 10.1016/j.brs.2025.02.022 40054574

[CR41] Mian OS, Li Y, Antunes A, Glover PM, Day BL (2013) On the vertigo due to static magnetic fields. PLoS ONE 8:e78748. 10.1371/journal.pone.0078748 PMC3813712 24205304

[CR42] Mittelstaedt H (1996) Somatic graviception. Biol Psychol 42:53–74. 10.1016/0301-0511(95)05146-5 8770370

[CR43] Napadow V, Sheehan JD, Kim J, LaCount LT, Park K, Kaptchuk TJ, Rosen BR, Kuo B (2013) The brain circuitry underlying the temporal evolution of nausea in humans. Cereb Cor 23:806–813. 10.1093/cercor/bhs073 PMC3593575 22473843

[CR44] Oppenheimer SM (1994) Neurogenic cardiac effects of cerebrovascular disease. Curr Opin Neurol 7:20–24. 10.1097/00019052-199402000-00005 8173672

[CR45] Oppenheimer SM, Gelb A, Girvin JP, Hachinski VC (1992) Cardiovascular effects of human insular cortex stimulation. Neurology 42:1727–1732. 10.1212/wnl.42.9.1727 1513461

[CR46] Ruffle JK, Patel A, Giampietro V, Howard MA, Sanger GJ, Andrews PL, Williams SC, Aziz Q, Farmer AD (2019) Functional brain networks and neuroanatomy underpinning nausea severity can predict nausea susceptibility using machine learning. J Physiol 597:1517–1529. 10.1113/JP277474 PMC6418775 30629751

[CR47] Stephan T, Deutschländer A, Nolte A, Schneider E, Wiesmann M, Brandt T, Dieterich M (2005) Functional MRI of galvanic vestibular stimulation with alternating currents at different frequencies. NeuroImage 26:721–732. 10.1016/j.neuroimage.2005.02.049 15955481

[CR48] Theysohn JM, Kraff O, Eilers K, Andrade D, Gerwig M, Timmann D, Schmitt F, Ladd ME, Ladd SC, Bitz AK (2014) Vestibular effects of a 7 Tesla MRI examination compared to 1.5 T and 0 T in healthy volunteers. PLoS ONE 9:e92104. 10.1371/journal.pone.0092104 PMC3962400 24658179

[CR49] von Gierke HE, Parker DE (1993) Differences in otolith and abdominal viscera graviceptor dynamics: implications for motion sickness and perceived body position. Aviat Space Environ Med 65:747–751 7980337

[CR50] Wade MG, Jones G (1997) The role of vision and spatial orientation in the maintenance of posture. Phys Ther 77:619–628. 10.1093/ptj/77.6.619 9184687

[CR51] Yates B, Miller A, Lucot J (1998) Physiological basis and pharmacology of motion sickness: an update. Brain Res Bull 47:395–406. 10.1016/S0361-9230(98)00092-6 10052567

[CR52] Zhong W, Shahbaz O, Teskey G, Beever A, Kachour N, Venketaraman V, Darmani NA (2021) Mechanisms of nausea and vomiting current knowledge and recent advances in intracellular emetic signaling systems. Int J Mol Sci 22:5797. 10.3390/ijms22115797 PMC8198651 34071460

[CR53] zu Eulenburg P, Caspers S, Roski C, Eickhoff SB (2012) Meta-analytical definition and functional connectivity of the human vestibular cortex. NeuroImage 60:162–169. 10.1016/j.neuroimage.2011.12.032 22209784

